# Testing a Smartphone App (Young with Diabetes) to Improve Self-Management of Diabetes Over 12 Months: Randomized Controlled Trial

**DOI:** 10.2196/mhealth.9487

**Published:** 2018-06-26

**Authors:** Pernille Castensøe-Seidenfaden, Gitte Reventlov Husted, Andreas Kryger Jensen, Eva Hommel, Birthe Olsen, Ulrik Pedersen-Bjergaard, Finn Kensing, Grete Teilmann

**Affiliations:** ^1^ Nordsjællands Hospital Pediatric and Adolescent Department University of Copenhagen Hillerød Denmark; ^2^ Institute of Public Health Biostatistics University of Copenhagen Copenhagen Denmark; ^3^ Nordsjællands Hospital Department of Clinical Research University of Copenhagen Hillerød Denmark; ^4^ Steno Diabetes Center, Copenhagen University of Copenhagen Gentofte Denmark; ^5^ Herlev Hospital Pediatric and Adolescent Department University of Copenhagen Herlev Denmark; ^6^ Nordsjællands Hospital Department of Cardiology, Nephrology, and Endocrinology University of Copenhagen Hillerød Denmark; ^7^ Department of Computer Science University of Copenhagen Copenhagen Denmark

**Keywords:** mHealth, randomized controlled trial, self-management, diabetes, young people, transition

## Abstract

**Background:**

Young people often struggle to self-manage type 1 diabetes during the transition from childhood to adulthood. Mobile health (mHealth) apps may have the potential to support self-management, but evidence is limited and randomized controlled trials are needed.

**Objective:**

We assessed whether the mHealth app “Young with Diabetes” improved young people’s self-management measured by glycated hemoglobin (HbA_1c_) and three self-reported psychometric scales.

**Methods:**

Young people (14-22 years) with inadequate glycemic control and their parents were enrolled in a randomized controlled trial and assigned either to Young with Diabetes and usual care (Young with Diabetes group) or to usual care alone (control). Young with Diabetes use was monitored; functions included a chat room, contact the health care provider, reminders, tips, information about the diabetes department and type 1 diabetes topics, carbohydrate counting, and a parents’ section. Outcomes included HbA_1c_ and three self-reported psychometric scales: Perceived Competence in Diabetes Scale; Health Care Climate Questionnaire; and Problem Areas In Diabetes care survey. Data were collected at baseline and at 2, 7, and 12 months.

**Results:**

A total of 151 young people were randomized (Young with Diabetes group=76, control=75) and 49 parents agreed to participate. At 12 months, HbA_1c_ was significantly higher (4.1 mmol/mol; 0.4 %) in the Young with Diabetes group, compared to the control group (*P*=.04); this finding did not occur when comparing app users (Young with Diabetes use ≥5 days) with nonusers. Young people used Young with Diabetes on a mean of 10.5 days. They spent the most time chatting about alcohol and searching for information about sex. Most young people and half of the parents reported that Young with Diabetes helped them. More than 80% would recommend Young with Diabetes to peers.

**Conclusions:**

Young with Diabetes did not improve HbA_1c_, but it may be a useful complement to self-management. Qualitative evaluation is needed to explore benefits and shortcomings of Young with Diabetes. Health care providers should address young peoples’ knowledge about sensitive topics, provide them with peer support, and be aware of parents’ need for information about how to support

**Trial Registration:**

ClinicalTrials.gov NCT02632383; https://clinicaltrials.gov/ct2/show/NCT02632383 (Archived by WebCite at http://www.webcitation.org/6zCK2u7xM)

## Introduction

### Background

As young people with type 1 diabetes (T1DM) grow up, they are expected to assume responsibility for their disease self-management [1]. This includes daily insulin dosage, glucose measurements, and carbohydrate counting to meet the recommended target for glycemic control [2]. However, young people often struggle to achieve adequate glycemic control [3], risking early onset of long-term complications [4]. Parents are key players in supporting young people in self-managing T1DM, but they are often faced with stress and frustration [5] and request guidance on how to support their children [6].

Self-management is defined as an individual’s ability to manage the symptoms and the consequences of living with a chronic condition, including treatment, physical, social, and lifestyle changes [7]. In young people, self-management is a gradual process of acquiring necessary skills and knowledge, with parents as consultants [1].

Mobile health (mHealth) apps present unique opportunities to engage young people in self-management by providing information and optimizing communication with health care providers [8]. Recent studies among adults show promising results. A systematic review assessed the effectiveness of self-management apps in long-term conditions and found that six of nine studies significantly improved outcomes [9]. Another systematic review of 12 randomized controlled trials (RCTs) demonstrated a significant reduction of glycated hemoglobin (HbA_1c_) in adults (particularly with type 2 diabetes) allocated to app-based interventions to support diabetes self-management [10].

However, limited evidence exists that mHealth apps can improve young peoples’ self-management [11]. Only three mHealth apps for young people with T1DM have been evaluated. Frøisland et al [12] tested a digital diabetes diary in a three-month prospective cohort study. At a mandatory consultation, the diary was discussed, and patients and providers reflected on its recordings (n=12; ages 13-19). Berndt et al [13] tested an app to collect data and provide clinical support in a four-week RCT (n=68; ages 8-18). Finally, Goyal et al [14] tested an mHealth app in a 12-month RCT (n=92; ages 12-15). The app facilitated feedback on the transfer of blood glucose readings from a glucometer, rewarding action. The three studies found no improvement in HbA_1c_ compared to the control group. However, one study [14] found a statistically significant association between increased self-monitored blood glucose and improved HbA_1c_. Unfortunately, comparability is limited by the small number of existing studies and differences in intervention design. As the number of mHealth apps rapidly increases, a pressing need arises for more RCTs to assess the impact of mHealth apps among young people and their parents [15].

### Young with Diabetes - The mHealth App

The mHealth app, Young with Diabetes (YWD), was developed in 2014 and 2015 in a mixed-methods design based on a participatory approach, with the aim of supporting young people and parents in T1DM self-management. Usability was tested in think-aloud tests and by a mail panel, and feasibility was tested for five weeks by young people and health care providers. The development is detailed elsewhere [16]. YWD is based on the premise that providing a platform for young people to access information and support from peers, parents, and health care providers will improve their self-management skills. YWD comprises eight main functions (Multimedia Appendix 1) described in the following: (1) *My Page* enables users to contact their health care provider and write notes, (2) *My Department* provides information about the diabetes department, (3) *Chat Room* is an opportunity to chat with peers, (4) *Carbohydrate Counting* provides information on how to count carbohydrates, (5) *Information about…* provides information about multiple T1DM-topics, such as obtaining a drivers’ license, (6) *Tips Package* enables users to receive daily T1DM tips, (7) *To Parents* provides parents with information about how to support their teen, and (8) *Reminder Function* allows users to set reminders for self-management tasks.

The aim of this study was to test whether YWD improved self-management, measured by HbA_1c_ and three psychometric scales, among young people with T1DM, compared with usual outpatient care.

## Methods

### Design, Sample, and Setting

A 12-month, open, parallel RCT was conducted. Young people were eligible for the study if they satisfied the following conditions: (1) they had been diagnosed with T1DM for more than one year, (2) received diabetes care at one of three pediatric or three adult outpatient clinics (Multimedia Appendix 2), (3) were 14 to 22 years of age, (4) had a HbA_1c_ ≥64 mmol/mol (8%) at their last visit and an average HbA_1c_>58 mmol/mol (7.5%) at the last three visits prior to invitation, (5) did not attend appointments with a psychiatrist or psychologist, (6) they spoke and understood Danish, and (7) did not participate in other diabetes intervention studies. Parents were invited to participate if their child was randomized to the YWD group and if they spoke and understood Danish.

### Recruitment Procedures

Young people and parents were recruited from November 2015 to March 2016. They received an invitation letter, followed by a phone call to answer any questions. If young people were interested, a one-hour meeting was scheduled to complete written consents and randomization. Participants were digitally randomized in a 1:1 allocation ratio either to YWD and usual care (YWD group) or usual care alone (control). They were stratified by department in random permuted blocks of two and four. Blinding was not possible.

### Intervention

After randomization, young people and parents downloaded YWD on their smartphone or tablet during a 10-minute initial face-to-face or telephone guidance session provided by the first author. The parents received the same version of YWD except for the *Chat Room*, which was only available for young people. Young people were encouraged to use YWD as a stand-alone resource and in collaboration with their parents and health care providers. They received no prompts to use YWD. The control group received only usual outpatient care, which consisted of quarterly clinic visits (measuring HbA_1c_, adjusting insulin and receiving guidance on carbohydrate counting).

Physicians, nurses, and dieticians provided the YWD intervention as part of usual outpatient care and saw participants from both the YWD and control groups. No extra time was allocated for the YWD intervention. Health care providers attended YWD training: a one-hour introduction to the app followed by two roleplaying scenarios with a colleague or the first author acting as young patients [16].

The first author offered monthly visits to health care providers to address technical issues and refresh training in app use; a telephone hotline was available for technical difficulties. The app content did not change during the study.

### Outcome Measures

Outcomes data were collected at baseline and two months, seven months, and 12 months after YWD use began. The primary outcome of HbA_1c_ was measured by a single automated glycohemoglobin analyzer (Tosoh) at Nordsjællands Hospital. Three psychometric self-reported scales measured the secondary outcome of the development of self-management skills. Perceived competence at managing diabetes was measured by the five-item Perceived Competence in Diabetes Scale (PCD) [17]. The degree to which participants experienced their health care provider to be autonomy-supportive in providing general treatment was measured using the five-item Health Care Climate Questionnaire (HCCQ) [17]. The perceived burden of diabetes-related problems was assessed using the 20-item Problem Areas in Diabetes care survey (PAID-20) [18]. Severe hypoglycemic episodes (low blood glucose levels requiring assistance from another person) and acute diabetes-related hospitalizations were self-reported.

### Sociodemographic Items and Young with Diabetes-Specific Questions

Sociodemographic characteristics (gender, age, height, weight, age at diabetes onset, occupation, family structure, comorbidity, insulin regime, weekly blood glucose measurements, transfer to adult care, smoking, and alcohol use) were self-reported. Responses to YWD-specific questions, such as “Has YWD helped you?” and “Would you recommend it to peers?” were self-reported using yes/no response options.

The psychometric scales, sociodemographic items, and YWD-specific questions were compiled into an electronic questionnaire. Face validity was tested in six young people before the trial start; no changes were required.

YWD users were defined as those who had used YWD on at least five days. The cutoff of five days was set to be sure the participants used the app more than the four times where they were paid a visit from the data collector (baseline, 2, 7, and 12 months). YWD use was documented by log data as time, date, and action (view, update, create, delete). Page hits were defined as the number of “clicks” within a function. Technical issues were noted.

### Power Estimation

Sample size estimation was based on HbA_1c_. A minimum of 52 participants per group was necessary to detect a difference of 5.5 mmol/mol (0.5 %) in HbA_1c_ at 80% power with 5% significance level, a standard deviation in the outcome variable of 0.5, and a 2-tailed significance test. To compensate for potential dropouts, a 25% adjustment was made, resulting in a target sample size of 65 subjects per group.

### Statistical Analysis

Baseline data were described by mean and standard deviation (continuous variables) and frequencies and proportions (categorical variables). In accordance with the CONSORT guidelines [19], hypothesis tests for baseline differences were not performed.

The primary intention-to-treat analysis, comparing groups at 12 months, was performed by a linear regression model adjusting for baseline values and diabetes department. Due to stratified randomization, the department was included in the regression model as a categorical covariate [20].

The effect of YWD depends on use. Consequently, the CONSORT-EHEALTH checklist [21] recommends a sub-group analysis comparing users with nonusers, equivalent to an as-treated analysis. YWD use is a post-randomization variable, and the possibility that several unmeasured factors affected both the probability of noncompliance with the intervention and glycemic control confounds the as-treated analysis. We, therefore, focused on estimating the complier average causal effect of YWD [22]. The analysis compared the effect of the intervention among compliers (the observed YWD users and those from the control group who would have been YWD users had they been assigned to the YWD group) and non-compliers (the observed YWD non-users and those from the control group who would have used YWD less than 5 days had they been assigned to the YWD group) [22]. The causal effect of YWD on HbA_1c_ at 12 months among compliers was estimated by the expectation-maximization algorithm assuming normally distributed outcomes in each of the principal strata under one-sided noncompliance. This estimate was adjusted for baseline HbA_1c_ and department. Baseline variables were included as covariates for the probability of compliance with the treatment allocation in a latent logistic regression model.

Secondary analyses of outcomes (HbA_1c_, PCD, HCCQ and PAID) over time were performed using a constrained mixed model incorporating all measurement periods [23]. Confidence intervals were calculated using normal approximation. The number of acute hospitalizations and severe hypoglycemic episodes was compared by logistic regression after dichotomizing outcomes into zero or one or more events.

Analyses were performed by a statistician blinded to group assignment using R version 3.3.3 and Mplus7. A value of *P* ≤.05 was considered to be statistically significant.

### Ethical Considerations

YWD complies with regulations for protecting personal health information. A code was required to access YWD in addition to user name and password. Written informed consent was obtained from young people and parents, and parental consent was required for participants younger than 18 years. The study was approved by the Danish Data Protection Agency (no. 04015 NOH-2015-031) and performed in accordance with ethical recommendations of Helsinki Declaration. Ethical approval by Research Ethics Committee was not necessary (Ref.no. 14013934). The study is registered at ClinicalTrials.gov (NCT02632383). The RCT is reported in accordance with the CONSORT-EHEALTH guidelines for improving and standardizing evaluation reports of Web-based and mobile health interventions (Multimedia Appendix 3 shows the CONSORT-EHEALTH checklist [21])

## Results

### Overview

**Figure 1:**
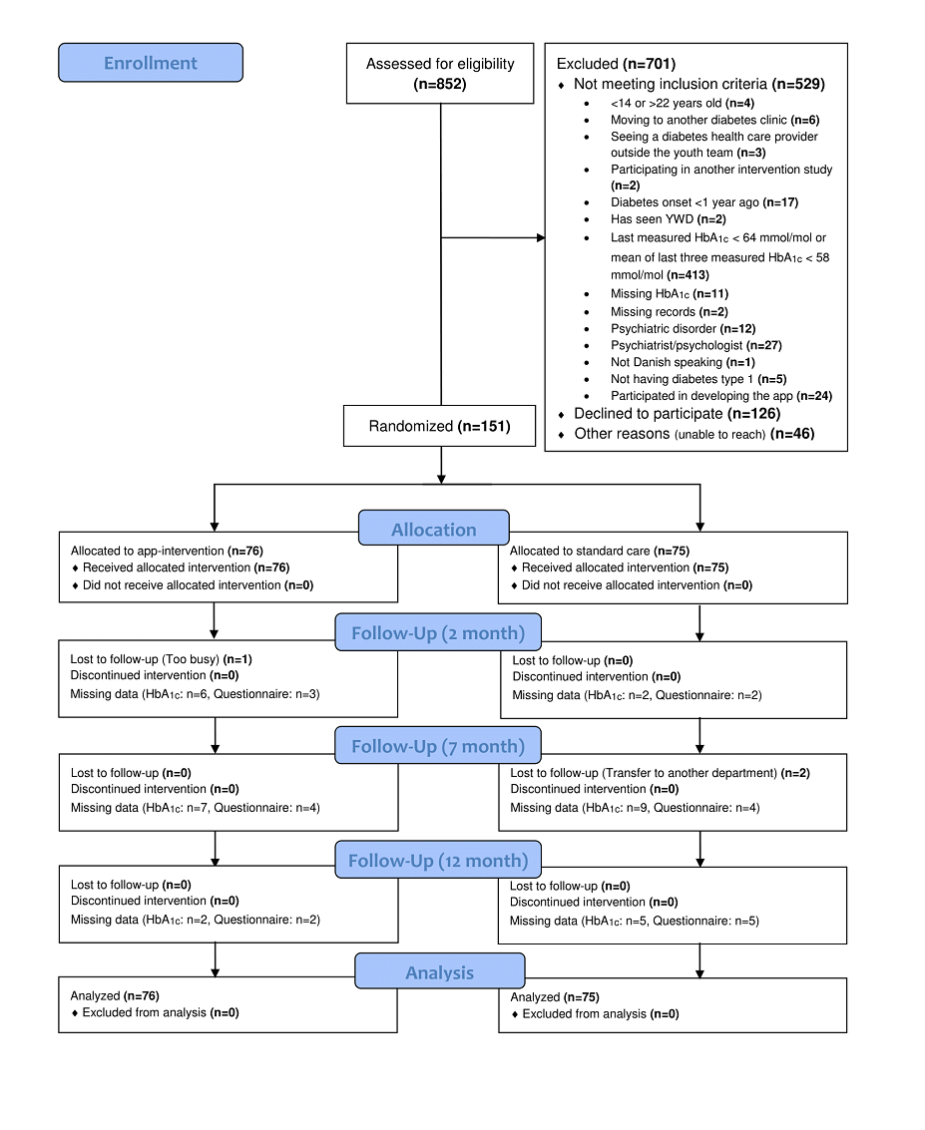
Participant flow diagram.

A total of 852 young people were assessed for eligibility, of whom 701 were excluded (Figure 1). In total, 126 young people declined to participate because they were too busy (n=64), were not interested in the research project (n=29), did not want to focus on diabetes (n=11), did not feel they needed the app (n=10), had no reason (n=9), or due to illness (n=3). A total of 151 young people (54% female) were randomized to the YWD group (n=76) or control group (n=75); of these, 148 (YWD=75, control=73) completed follow-up assessments, yielding a retention rate of 98%.

Participants were enrolled at their homes (n=121), school (n=10), hospital (n=9), café (n=4) or by phone (n=7).

### Baseline Characteristics

Participants’ mean age was 17.6 (SD 2.6) years, and their mean duration of T1DM was 8.0 (SD 4.5) years (Table 1). One third (n=42, 28%) had at least one comorbidity, and half (n=70, 46%) of the participants’ parents were divorced. A total of 49 parents participated, representing 40 (53%) young people in the YWD group.

### Outcome Measures

#### Glycated Hemoglobin

**Figure 2:**
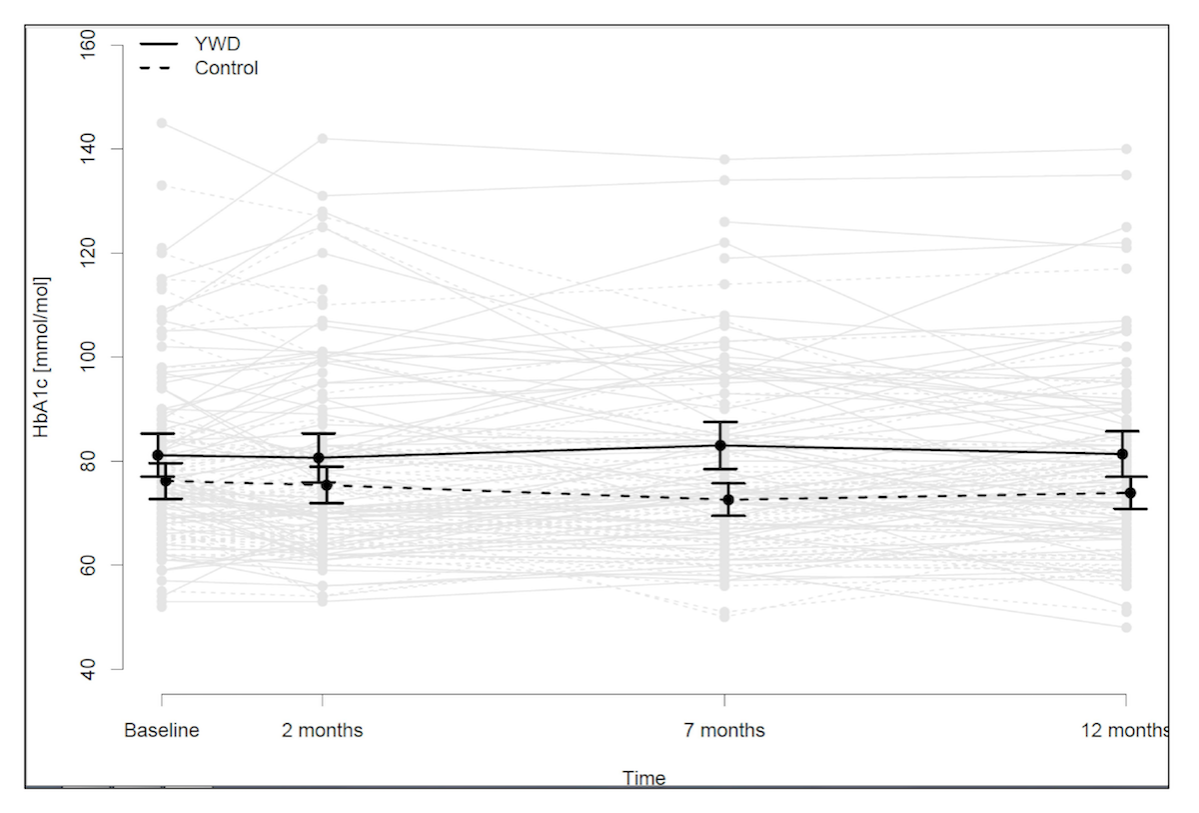
Mean glycated hemoglobin (HbA_1c_) levels in control and Young with Diabetes (YWD) groups at 2, 7, and 12 months.

Mean baseline HbA_1c_ (Figure 2 and Table 2) was 81.1 mmol/mol (SD 18.0) or 9.6% (SD 1.6) in the YWD group and 76.2 mmol/mol (SD 14.9) or 9.1% (SD 1.4) in the control group. This difference was not significant (*P*=.07). At the 12-month follow-up, mean HbA_1c_ was 81.4 mmol/mol (SD 18.8) or 9.6% (SD 1.7) in the YWD group and 73.9 mmol/mol (SD 12.6) or 8.9% (SD 1.2) in the control group. The intention-to-treat-analysis, comparing the two groups at 12 months, showed a significant difference in glycemic control (*P*=.04), with the control group having a 4.1 mmol/mol (95% CI 0.3-7.9) or 0.4% (95% CI 0.0-0.7) lower mean HbA_1c_ after adjusting for baseline values. After including all follow-up periods in the mixed model, this difference was 4.3 mmol/mol (95% CI 0.7-8.0) or 0.4% (95% CI 0.1-0.7, *P*=.02). Despite randomization, the YWD group included more females. This difference was not significant (*P*=.37); adjusting for gender in the intention-to-treat analysis did not change the results.

#### Effect of App Use on Glycated Hemoglobin

The as-treated analysis, comparing YWD users with nonusers, yielded a non-significant difference in HbA_1c_ at 12 months (*P*=.67), with the control group having a 0.9 mmol/mol (95% CI –3.1 to 4.9) or 0.1% (95% CI –0.3 to 0.4) lower mean HbA_1c_. The complier average causal effect of YWD, comparing the effect of the intervention among compliers and non-compliers (please refer to the Statistical Analysis section for further details), yielded a non-significant difference of 3.9 mmol/mol (95% CI –0.7 to 8.9) or 0.4% (95% CI –0.1 to 0.8, *P*=.11) in HbA_1c_, favoring the control group. No baseline covariates were significantly associated with the probability of compliance with the treatment allocation (Multimedia Appendix 4). However, a negative effect on the probability of compliance to the treatment allocation was related to comorbidity, divorced parents, severe hypoglycemic episodes during the previous 12 months, forgetting insulin, smoking, alcohol-drinking intake and skipping school. A positive effect on the probability of compliance with the treatment allocation was related to the number of glucose measurements last week, acute hospitalizations, insulin pump and the female gender.

#### Self-Reported Self-Management of Type 1 Diabetes

As shown in Table 2, no significant effects on PCD (*P*=.39), PAID (*P*=.13), or HCCQ (*P*=.53) were observed.

#### Hypoglycemia and Hospitalizations

Between-group differences in acute diabetes-related hospitalizations and severe hypoglycemia were not statistically significant. Seventeen (22%) participants from the YWD group and 8 (11%) participants from the control group were hospitalized for an acute event at least once during the 12-month study period. The control group had 54% lower odds (odds ratio [OR] 0.46, 95% CI 0.17-1.15, *P*=.10) of acute hospitalization after adjusting for acute hospitalizations during the 12 months prior to enrollment. A total of 34 (45%) participants in the YWD group and 29 (39%) in the control group experienced at least one episode of severe hypoglycemia. The control group had 13% lower odds (OR 0.87, 95% CI 0.43-1.75, *P*=.70) of severe hypoglycemia, compared to YWD group, after adjusting for hypoglycemic episodes during the year prior to the study.

**Table 1:** Sample characteristics.

Characteristics		YWD^a^ (n=76)	Control (n=75)
Female, n (%)		44 (58)	37 (49)
Age in years, mean (SD)		17.6 (2.6)	17.6 (2.7)
Age at diabetes onset in years, mean (SD)		9.2 (4.3)	9.9 (4.9)
Diabetes duration in years, mean (SD)		8.3 (4.3)	7.7 (4.7)
Baseline HbA_1c_^b^ (mmol/mol), mean (SD)		81.1 (18.0)	76.2 (14.9)
Baseline PCD^c^ score, mean (SD)		27.4 (6.0)	27.5 (6.2)
Baseline PAID^d^ score, mean (SD)		26.7 (19.3)	24.0 (16.1)
Baseline HCCQ^e^ score, mean (SD)		28.2 (6.8)	25.4 (8.4)
≥1 acute diabetes-related hospital admission^f^, n (%)		19 (25)	13 (17)
≥1 episodes of severe hypoglycemia, n (%)		27 (36)	23 (31)
SMBG^g^ per week, mean (SD)		24.4 (12.8)	25.8 (15.5)
**Forget to take insulin, n (%)**			
	Every day	5 (7)	10 (13)
	One to four times a week	24 (32)	24 (32)
	One or more times a month	25 (33)	22 (29)
	Never or almost never	22 (29)	19 (25)
BMI^h^, kg/m^2^, mean (SD)		22.1 (3.2)	23.3 (3.4)
Smoking cigarettes ≥1 time in the last month, n (%)		23 (30)	25 (33)
Drinking alcohol ≥1 time in the last month, n (%)		50 (66)	50 (67)
**Insulin regimen, n (%)**			
	Multiple daily injections of insulin	40 (53)	40 (53)
	Pump	36 (47)	35 (47)
Living with both parents, n (%)		34 (45)	32 (43)
Divorced parents, n (%)		38 (50)	32 (43)
**Education, n (%)**			
	Danish public school (grade 0-10)	28 (37)	26 (35)
	Continuation school	2 (3)	2 (3)
	Secondary education^i^	15 (20)	14 (19)
	University	6 (8)	7 (9)
	Other schools^j^	12 (16)	10 (13)
	Not attending a school at the moment	13 (17)	16 (21)
**Pediatric site, n (%)**			
	Pediatric and Adolescent Department, Nordsjællands Hospital, Hillerød	12 (16)	13 (17)
	Pediatric and Adolescent Department, Herlev	26 (34)	26 (35)
	Pediatric Department, Roskilde	7 (9)	7 (9)
**Adult site, n (%)**			
	Department of Cardiology, Nephrology and Endocrinology, Nordsjællands Hospital, Hillerød	6 (8)	6 (8)
	Steno Diabetes Center	20 (26)	20 (27)
	Department of Endocrinology, Køge	5 (7)	3 (4)
Transfer to adult care, n (%)		7 (9)	5 (7)
**Comorbidity, n (%)**		22 (29)	20 (27)
	Learning disability and/or mental health condition	6 (8)	2 (3)

^a^YWD: Young with Diabetes.

^b^HbA_1c_: glycated hemoglobin.

^c^PCD: Perceived Competence in Diabetes Scale.

^d^PAID: Problem Areas in Diabetes Scale.

^e^HCCQ: Health Care Climate Questionnaire.

^f^Acute hospital admission caused by hyperglycemia, ketoacidosis or hypoglycemia.

^g^SMBG: self-monitored blood glucose.

^h^BMI: body mass index.

^i^Secondary education: Gymnasium, Higher Preparatory Examination, Higher Commercial Examination Program, Higher Technical Examination Program.

^j^Other schools, such as taking a bachelor in nursing or attending a school of crafts.

**Table 2:** Between-group differences in outcomes.

Outcome	Adjusted mean at baseline	Control versus YWD^a^, mean difference (95% CI)			*P* value^b^
		2 months	7 months	12 months	
HbA_1c_^c^, mmol/mol	78.9	–2.8 (–5.4 to –0.3)	–6.2 (–9.5 to –2.9)	–4.3 (–8.0 to 0.7)	0.02
HbA_1c_^c^, %	9.4	–0.3 (–0.5 to 0.0)	–0.6 (–0.9 to –0.3)	–0.4 (–0.7 to 0.1)	0.02
PCD^d^ score^e^	28.18	0.27 (–1.50 to 2.03)	–0.53 (–2.55 to 1.50)	–0.79 (–2.56 to 0.98)	0.39
PAID^f^ score^e^	23.68	–2.64 (–6.17 to 0.88)	0.96 (–3.00 to 4.91)	–3.14 (–7.22 to 0.95)	0.13
HCCQ^g^ score^e^	27.10	–0.05 (–2.44 to 2.35)	0.04 (–2.52 to 2.61)	–0.73 (–2.98 to 1.52)	0.53

^a^YWD: Young with Diabetes.

^b^Significance level of difference at 12 months follow-up.

^c^HbA_1c_: glycated hemoglobin.

^d^PCD: Perceived Competence in Diabetes Scale.

^e^Range for PCD and HCCQ is 5-35 and the range for PAID is 0-100.

^f^PAID: Problem Areas in Diabetes.

^g^HCCQ: Health Care Climate Questionnaire.

#### Young with Diabetes-Specific Questions

Fifty-nine (78%) young people and 25 (51%) parents reported that YWD had helped them at least once. Most young people (n=65, 85%) and parents (n=41, 84%) reported that they would recommend YWD to peers.

#### Young with Diabetes Use

**Figure 3:**
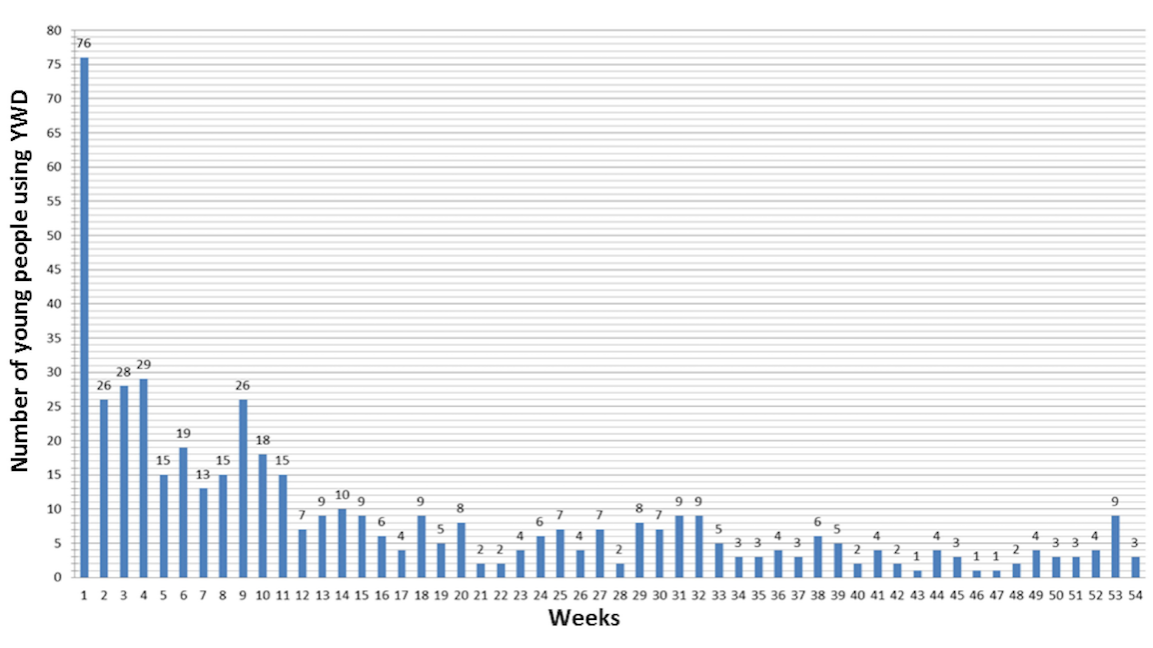
Number of young people who used Young with Diabetes (YWD) during the study.

Young people used YWD on a mean of 10.5 days (range 1-64), while parents used YWD on a mean of 5 days (range 1-21). A total of 53 (70%) young people and 19 (39%) parents used YWD on at least 5 days, while 7 (9%) young people and 13 (27%) parents never used YWD after the introductory session. Figure 3 depicts weekly YWD activity.

In total, 71 messages were sent to 14 (36%) health care providers by 15 (20%) young people. The messages were primarily used to schedule visits (n=25), ask treatment questions such as about insulin dose (n=24); discuss challenges such as eating disorders and feeling alone (n=9); and provide ongoing support such as feedback on glucose measurements (n=13).

A total of 103 chat-room comments were posted by 28 (37%) young people (Multimedia Appendix 5). The majority of chat time was spent on *Alcohol, Sport,* and *Fuck Diabetes*. Fifteen (20%) young people created reminders, and 46 (61%) activated tips packages. The carbohydrate-counting quiz was initiated 68 times by 46 (61%) young people. Only 7 (9%) young people watched animations, while 18 (24%) clicked on video self-portraits. The most popular main functions were *Chat Room* and *My Page* (Multimedia Appendix 6), and the most popular information topics were *Sex*, *What is Diabetes?, Driver’* *s License*, and *Alcohol and Party* (Multimedia Appendix 7). Among parents, the most popular main functions were *Information about* … and *To Parents* (Multimedia Appendix 6). Parents primarily approached *How to Support My Teen*, *When My Teen turns 18*, *Alcohol and Party*, and *Being Young with Diabetes* (Multimedia Appendix 7)*.*

### Technical Issues

Four major platform-specific technical issues occurred and were resolved: (1) January 2016, Android. Starting carbohydrate-counting-quiz resulted in log-off (duration=10 days, n=1), (2) March 2016, iOS. YWD could not open on some iPhone-software versions. Required re-installation (duration=10 days, n=7), (3) September 2016, Android. Unable to upload photos (duration=40 days, n=1), and (4) January 2017, iOS. YWD could not open due to update. Needed re-installation (duration=10 days, n=14). In addition, participants reported minor technical issues, such as having lost the YWD app due to new or broken phones (young people=26, parents= 2). A total of 43 (57%) young people and eight (16%) parents reported technical issues.

## Discussion

### Principal Findings

To the best of our knowledge, this is the largest RCT to date evaluating the effect of an mHealth app supporting self-management in young people with T1DM and their parents. YWD did not improve glycemic control, and the app use declined rapidly. Interestingly, most of the participants reported that YWD was helpful and that they would recommend it to others.

We can only speculate as to why HbA_1c_ did not improve in the YWD group. A large difference was observed between the results from the as-treated analysis and the estimate of the complier average causal effect (0.9 mmol/mol vs. 3.9 mmol/mol, respectively). This may indicate the existence of unmeasured confounding variables influencing HbA_1c_ and YWD use. Health care providers play a significant role in supporting young people to self-manage [24]. However, not all health care providers feel confident using mHealth apps [25], and some may feel uncomfortable engaging with young people through technical means [26,27]. This could have influenced the effect of YWD. Unfortunately, we neither registered the young people’s health care provider nor stratified at the level of the health care provider. Also, YWD training for health care providers was very brief; further training may optimize health care providers’ ability to use YWD as a platform for collaborating with young people and parents. Furthermore, the use of YWD declined rapidly during the RCT (Figure 3), which may be one of the main reasons why the intervention lacked improvement of self-management. Since the participants did not use YWD for long, a mediation analysis would have been highly relevant. However, the study was an RCT designed and powered for assessing the difference in HbA_1c_ and therefore, we did not pursue a post-hoc analysis. This is important to address in the design of future studies.

A qualitative study by Klasnja et al [28] found that most people diagnosed with diabetes, face acute need for information about their disease and that this need becomes more intermittent afterwards. It would have been highly relevant to test YWD in a group of people newly diagnosed with T1DM. Unfortunately, HbA_1c_ differs and changes a lot during the time around diagnosis depending on how long (days, weeks, or months) people have had diabetes before it is diagnosed and depending on the degree of the eventual honeymoon phase. Since HbA_1c_ was our primary outcome, we had to be sure that we only included patients with “stable” diabetes to better identify the effect of the intervention. This challenge could be addressed in future studies by qualitative evaluation of self-management apps in people just diagnosed with diabetes.

We were unable to measure participants’ eHealth skills, which may have influenced YWD use and subsequent HbA_1c_ levels since it is related to improved outcomes [29-31]. Furthermore, baseline HbA_1c_ was higher in the YWD group, which may indicate poor motivation and lack of self-management skills, which would affect the ability to use YWD and improve HbA_1c_[32]. Finally, it is arguable whether a randomized trial is the optimal way to evaluate YWD. Diabetes care should be individualized [33], and mHealth apps, which evolve and are updated over time, are often incompatible with a rigid RCT study design. Furthermore, Campbell et al [34] raise doubts about RCTs as an evaluation method targeting young people in transition from childhood to adulthood due to the complex, patient-centered, evolving, and multidisciplinary nature of care. Alternative methods may be preferable, such as qualitative evaluations and interrupted time series [35].

Further qualitative evaluation [36] is needed to understand why most young people reported being helped by using YWD, despite failing to improve glycemic control and maintain app use. Also, successful adoption of self-management apps is hard to achieve without additional strategies for enhancing patient motivation and engaging health care providers [37]. Finally, simply knowing how often and how much young people engage with YWD by opening the app and clicking around may not be enough. Understanding and observing “effective engagement” [38,39] with mHealth apps is much harder to do, and better ways need to be worked out. This should be taken into account in future studies.

Notably, the most popular app function among young people was the *Chat room*, where they shared experiences. The most popular topics were *Alcohol* and *Fuck Diabetes*. While few participants posted comments, most read about others’ experiences. This is consistent with previous findings [40,41] and underscores the importance of online peer support to complement education and provide reassurance that lived experiences are common [42].

In contrast, more sensitive topics, such as sex, were not discussed in the chat room but were the most popular topic searched privately. Wiley et al [42] explored young adults’ experiences with T1DM education and found that health care providers did not address sensitive topics such as sex. Our findings and those of Wiley et al highlight the unmet needs of young people and parents, which should be solicited and addressed regularly in clinic visits. They underscore the importance of acknowledging young peoples’ need for sharing experiences with peers and providing them with opportunities to engage with peer networks. The findings also emphasize parents’ need for guidance in supporting their child and the importance of addressing sensitive topics regularly.

### Strengths and Limitations

Our study has several strengths. A rigorous design tested YWD in an RCT over a lengthy study period, and YWD use was logged and available for analysis. Our study had both a large sample size and a high retention rate. The high retention rate could be a result of the flexibility to collect data at young peoples’ choice of place and time of day and should be considered a way to ensure high retention rates in future studies with young people.

Limitations should also be considered. It was not possible to conduct a blinded RCT [43,44]. Not all young people had participating parents. No clear criteria were defined for how health care providers should deliver YWD. Also, we cannot exclude the possibility of a spillover effect because the same health care professionals provided both the YWD intervention and usual care. Finally, a concern is whether HbA_1c_ and the three psychometric questionnaires (PCD, HCCQ, PAID-20) captured changes in self-management as intended. Our choice of scales was limited by lack of validated self-management instruments in Danish and also by the ages of the participants, spanning below and above 18 years. The outcomes were chosen based on the self-management definition [1,7] and because they have been used in similar populations testing self-management interventions [45,46], increasing the comparability of our study.

### Conclusion

The mHealth app YWD did not improve HbA_1c_, but it may be a useful tool for complementing self-management in young people with T1DM. Qualitative evaluation is needed to further explore and address benefits and shortcomings of the intervention [36]. Alternative evaluation methods should be considered when testing self-management mHealth apps among young people. Our findings highlight the importance of supplementing self-management care with peer support. Health care providers should routinely address sensitive topics and be aware of parents’ need for guidance as to how to effectively support their child during the transition from childhood to adulthood.

## Appendix

Multimedia Appendix 1 Detailed description of the mHealth application Young with Diabetes.

Multimedia Appendix 2 Diabetes departments participating in the RCT.

Multimedia Appendix 3 CONSORT-EHEALTH checklist (v1.6.1).

Multimedia Appendix 4 Association between baseline covariates and compliance.

Multimedia Appendix 5 Use of chat room groups.

Multimedia Appendix 6 Use of main functions by young people and parents.

Multimedia Appendix 7 Use of information topics by young people and parents.

## Abbreviations

HbA _1c_: glycated hemoglobin
HCCQ: Health Care Climate Questionnaire
OR: odds ratio
PAID: Problem Areas in Diabetes
PCD: Perceived Competence in Diabetes
RCT: randomized controlled trial
T1DM: type 1 diabetes
YWD: Young with Diabetes
