# Supplementary figures and images for: Testing a Smartphone App (Young with Diabetes) to Improve Self-Management of Diabetes Over 12 Months: Randomized Controlled Trial
Source: JMIR Mhealth Uhealth. 2018 Jun 26;6(6):e141. doi: 10.2196/mhealth.9487 (PMC6039771; doi:10.2196/mhealth.9487)

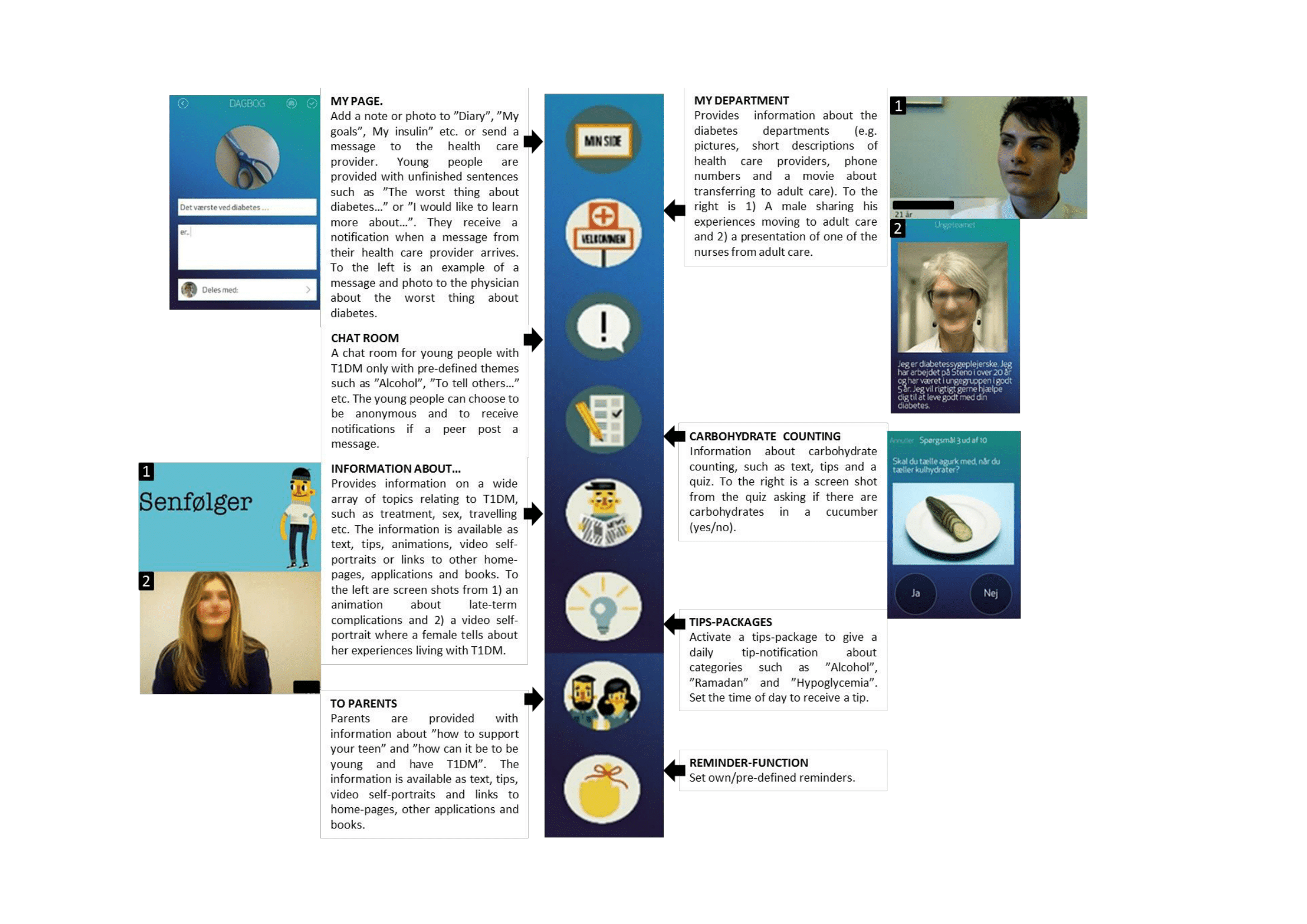

Supplement: Multimedia Appendix 1 [file mhealth_v6i6e141_app1.png]

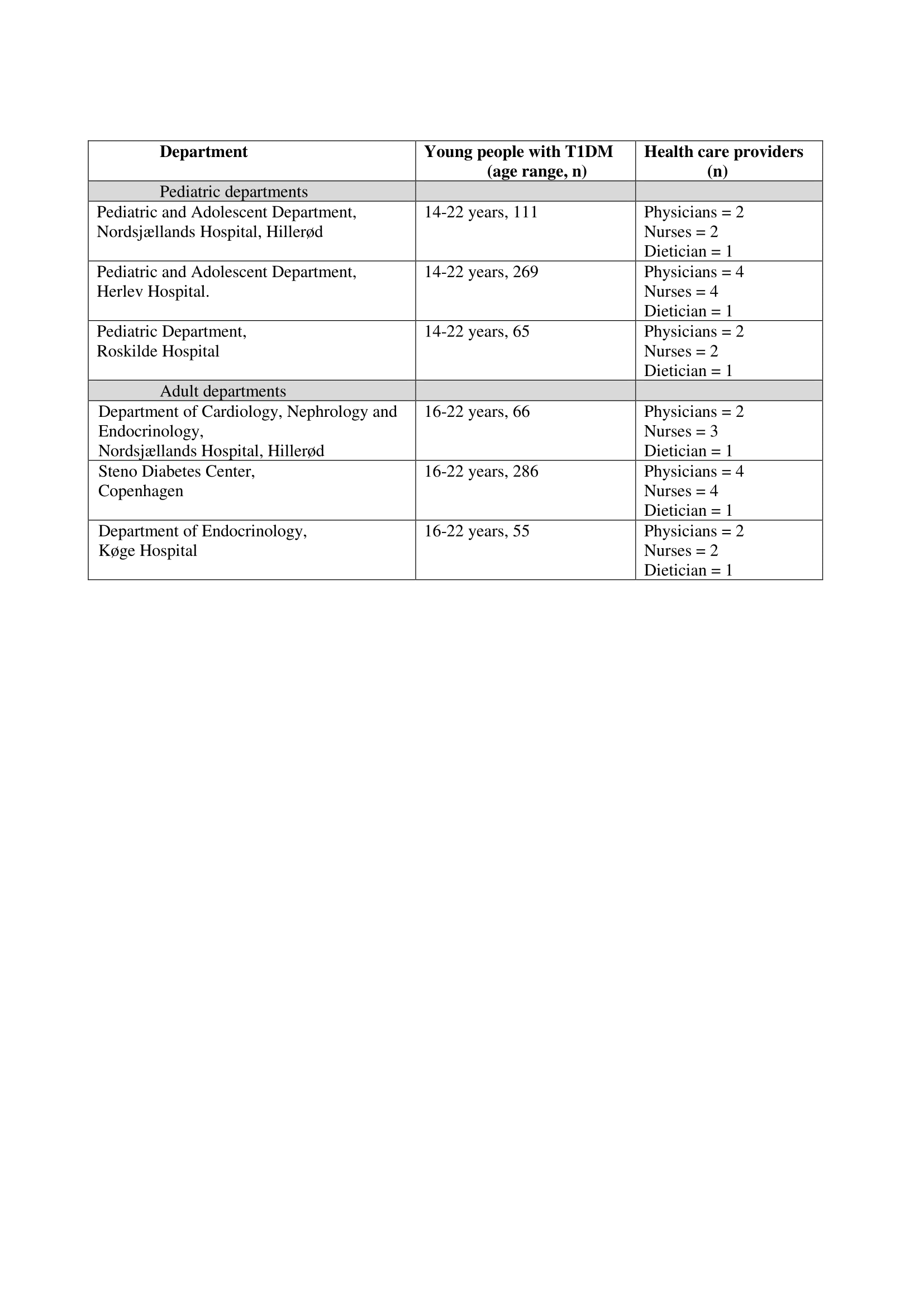

Supplement: Multimedia Appendix 2 [file mhealth_v6i6e141_app2.png]

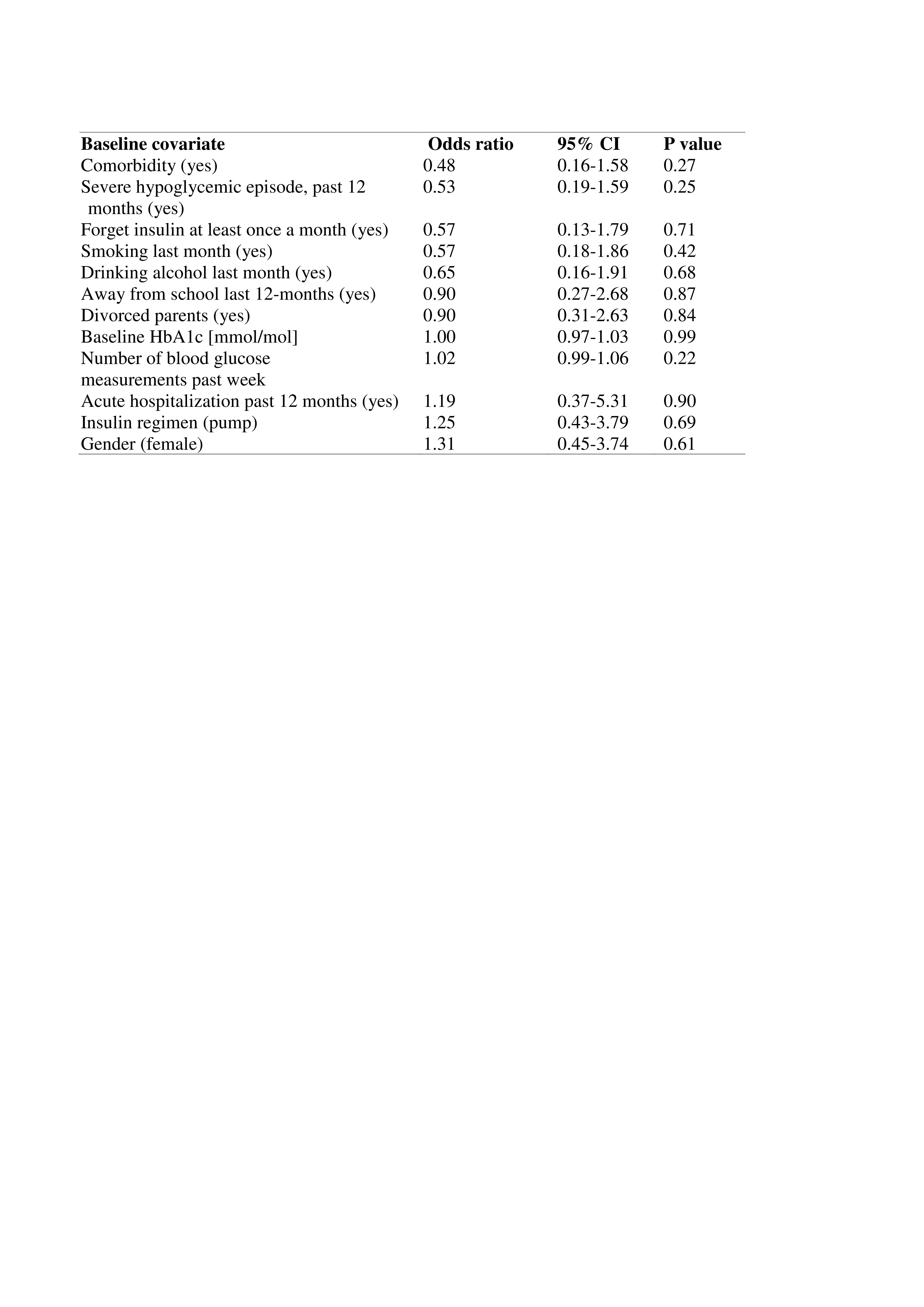

Supplement: Multimedia Appendix 4 [file mhealth_v6i6e141_app4.jpg]

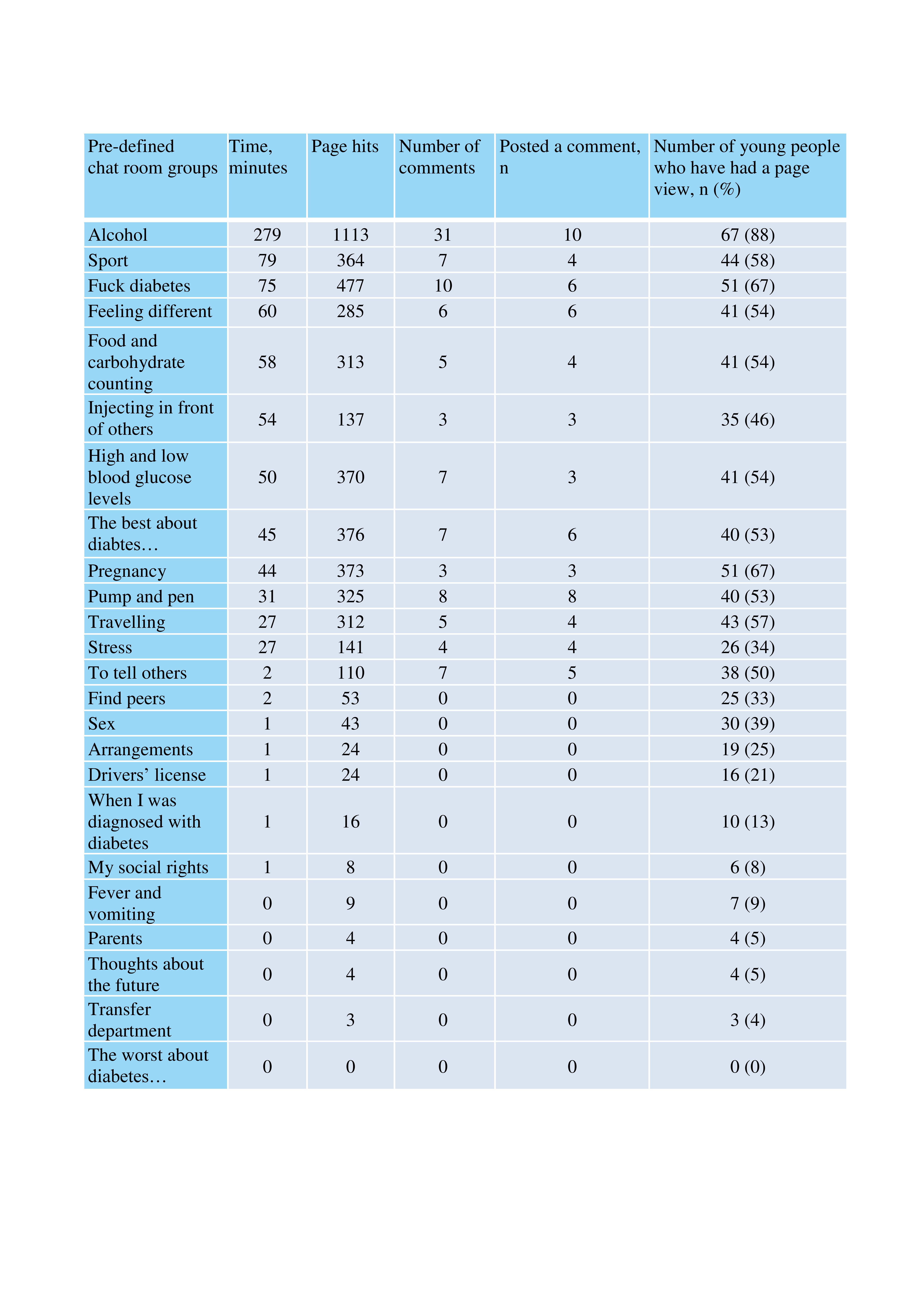

Supplement: Multimedia Appendix 5 [file mhealth_v6i6e141_app5.jpg]

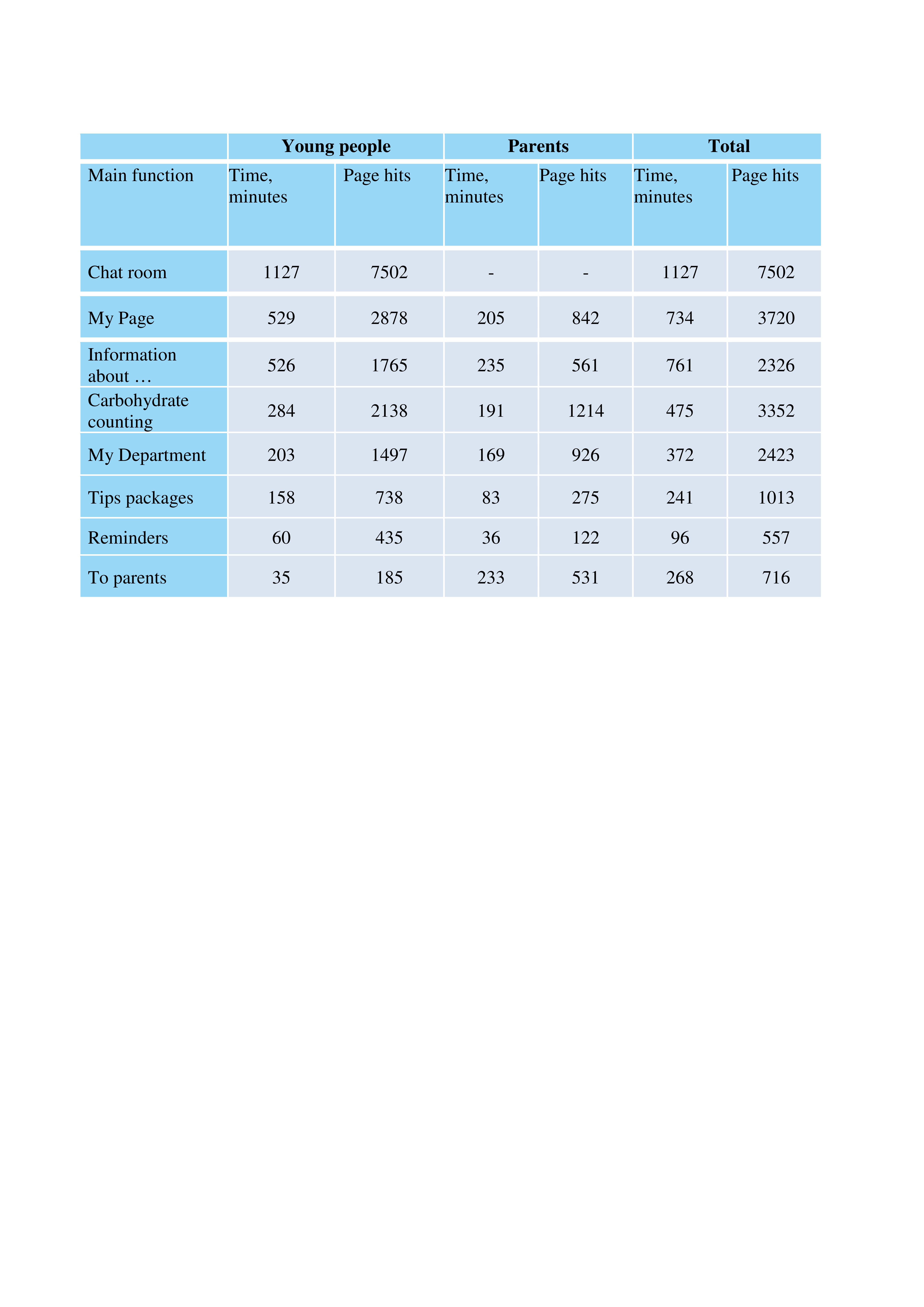

Supplement: Multimedia Appendix 6 [file mhealth_v6i6e141_app6.jpg]

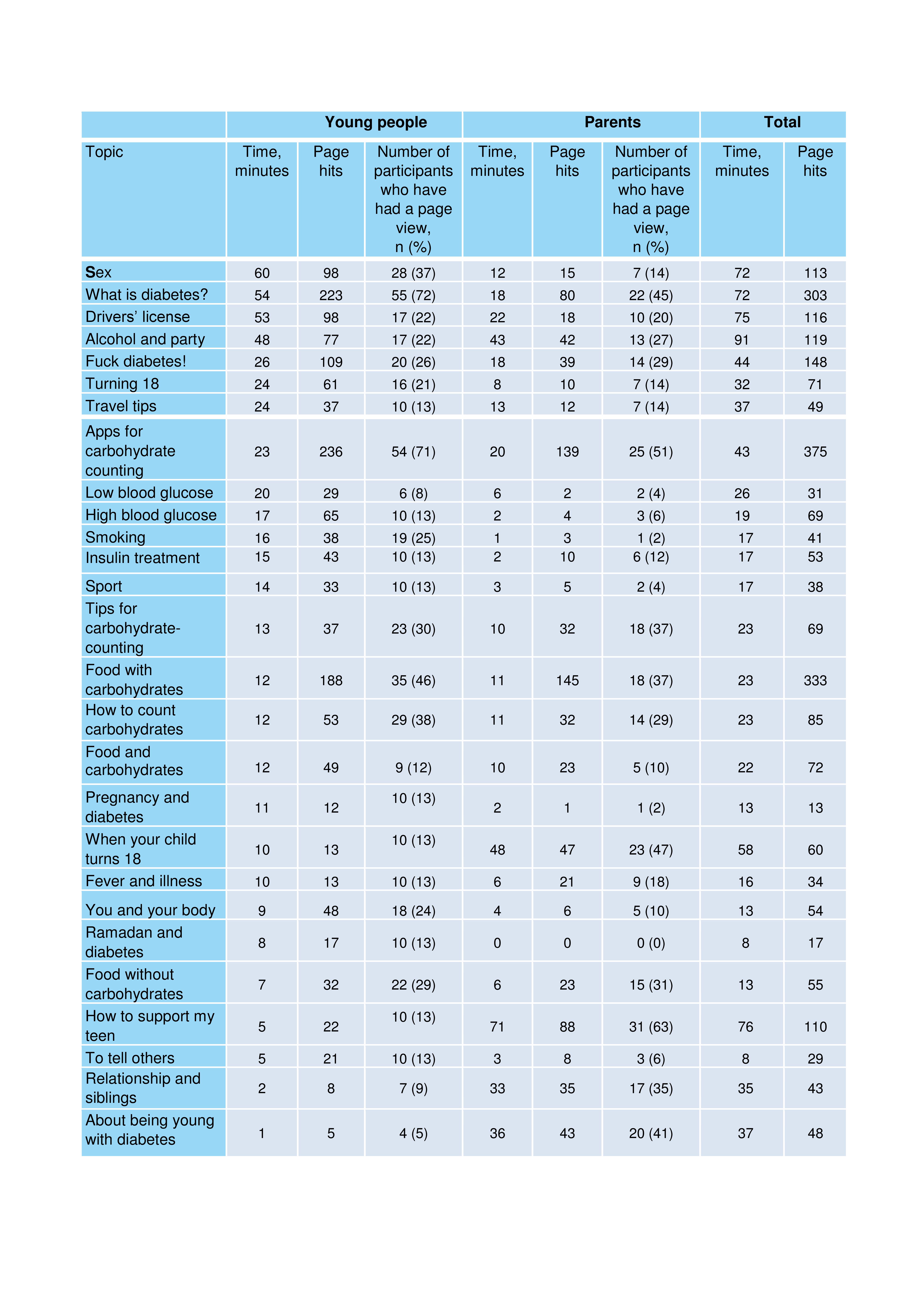

Supplement: Multimedia Appendix 7 [file mhealth_v6i6e141_app7.jpg]
