# Supplementary material for: Testing a Smartphone App (Young with Diabetes) to Improve Self-Management of Diabetes Over 12 Months: Randomized Controlled Trial
Source: JMIR Mhealth Uhealth. 2018 Jun 26;6(6):e141. doi: 10.2196/mhealth.9487 (PMC6039771; doi:10.2196/mhealth.9487)
Supplement: Multimedia Appendix 3 [file mhealth_v6i6e141_app3.pdf]

CONSORT-EHEALTH Checklist V1.6.2 Report	Manuscript Number	9487
**(based on CONSORT-EHEALTH V1.6), available at [http://tinyurl.com/consort-ehealth-v1-6].**		
**Date completed**		
6/11/2018 17:49:50		
**by**		
Pernille Castensøe-Seidenfaden		
Testing a smartphone app (Young with Diabetes) to improve self-management of diabetes over 12-months: Randomized controlled trial.		
TITLE		
**1a-i) Identify the mode of delivery in the title**		
"smartphone app"		
**1a-ii) Non-web-based components or important co-interventions in title**		
**1a-iii) Primary condition or target group in the title**		
"Young with Diabetes", "diabetes"		
ABSTRACT		
**1b-i) Key features/functionalities/components of the intervention and comparator in the METHODS section of the ABSTRACT**		
"...assigned either to YWD and usual care (YWD group) or to usual care alone (control)"<br><br>"...functions included a chat room, contact the health care provider, reminders,<br>tips, information about the diabetes department and type 1 diabetes topics, carbohydrate counting, and a parents' section."		
**1b-ii) Level of human involvement in the METHODS section of the ABSTRACT**		
"Young with Diabetes and usual care"		
**1b-iii) Open vs. closed, web-based (self-assessment) vs. face-to-face assessments in the METHODS section of the ABSTRACT**		
"... assigned either to Young with Diabetes and usual care (Young with Diabetes group) or usual care alone (control)"<br><br>"self-reported psychometric scales"		
**1b-iv) RESULTS section in abstract must contain use data**		
"A total of 151 young people were randomized (YWD group=76, control=75)"<br><br>"...HbA1c was significantly higher (4.1 mmol/mol [0.4 %]) in the YWD group, compared to the control group (P = .04)"<br><br>"...used YWD on a mean of 10.5 days."<br><br>"...spent the most time chatting about alcohol and searching for information about sex"		
**1b-v) CONCLUSIONS/DISCUSSION in abstract for negative trials**		
INTRODUCTION		
**2a-i) Problem and the type of system/solution**		

Description of the problem: Transition from childhood to adulthood, self-management of T1DM (please, refer to the introduction section)<br><br>Description of the solution: A description of the mHealth app Young with Diabetes (please, refer to the introduction section)<br><br>Goal of the intervention (please, refer to the aim)		
**2a-ii) Scientific background, rationale: What is known about the (type of) system**		
"Mobile health (mHealth) apps present unique opportunities to<br>engage young people in self-management by..."<br><br>"Only three mHealth apps for young people with T1DM have been evaluated..."<br><br>Please, refer to the introduction section for further details.		
**Does your paper address CONSORT subitem 2b?**		
"The aim of this study was to test whether YWD improved self-management, measured by HbA1c and three psychometric scales, among young people with T1DM, compared with usual outpatient care."		
METHODS		
**3a) CONSORT: Description of trial design (such as parallel, factorial) including allocation ratio**		
"A 12-month, open, parallel RCT..."<br><br>"...1:1 allocation ratio"		
**3b) CONSORT: Important changes to methods after trial commencement (such as eligibility criteria), with reasons**		
No important changes after trial commencement		
**3b-i) Bug fixes, Downtimes, Content Changes**		
Bug fixes and downtimes are described in details in the results section.		
**4a) CONSORT: Eligibility criteria for participants**		
"Young people were eligible for the study if they satisfied the following<br>conditions: (1) they had been diagnosed with T1DM for more than one year, (2) received diabetes care at one of three pediatric or three adult outpatient clinics ..., (3) were 14 to 22 years of age, (4) had a HbA1c ≥64 mmol/mol (8%) at their last visit and an average HbA1c>58 mmol/mol (7.5%) at the last three visits prior to invitation, (5) did not attend appointments with a psychiatrist or psychologist, (6) they spoke and understood Danish, and (7) did not participate in other<br>diabetes intervention studies."		
**4a-i) Computer / Internet literacy**		
Unfortunately this study did not address computer/internet literacy, since no validated scales were available in Danish.		
**4a-ii) Open vs. closed, web-based vs. face-to-face assessments:**		
"... (participants) received an invitation letter, followed by a phone call to answer any questions. If young people were interested, a one-hour meeting was scheduled to complete written consents and randomization."<br><br>"Physicians, nurses, and dieticians provided the YWD intervention as part of usual outpatient care..."		
**4a-iii) Information giving during recruitment**		
"... (participants) received an invitation letter, followed by a phone call to answer any questions."		
**4b) CONSORT: Settings and locations where the data were collected**		
"Data collected at "young peoples' choice of place and time of day"."		
**4b-i) Report if outcomes were (self-)assessed through online questionnaires**		

Secondary outcomes were (self-)assessed through electronic questionnaire (Please, refer to the methods section).		
**4b-ii) Report how institutional affiliations are displayed**		
**5) CONSORT: Describe the interventions for each group with sufficient details to allow replication, including how and when they were actually administered**		
**5-i) Mention names, credential, affiliations of the developers, sponsors, and owners**		
Conflicts of Interest:<br>"YWD was developed in cooperation with the IT enterprise Mobile Fitness A/S and the project group (including the authors). The project group owns the national rights."		
**5-ii) Describe the history/development process**		
"The mHealth app, Young with Diabetes (YWD), was developed in 2014 and 2015 in a mixed-methods design based on a participatory approach, with the aim of supporting young people and parents in T1DM self-management. Usability was tested in think-aloud tests and by a mail panel, and feasibility was tested<br>for five weeks by young people and health care providers. The development is detailed elsewhere [ref]."		
**5-iii) Revisions and updating**		
"The app content did not change during the study."		
**5-iv) Quality assurance methods**		
"The development is detailed elsewhere [ref]."		
**5-v) Ensure replicability by publishing the source code, and/or providing screenshots/screen-capture video, and/or providing flowcharts of the algorithms used**		
Please, find description of YWD in the Introduction section, including further details in the Multimedia Appendix.		
**5-vi) Digital preservation**		
**5-vii) Access**		
"... downloaded YWD on their smartphone or tablet during a 10-minute initial<br>face-to-face or telephone guidance session provided by the first author."<br><br>"A code was required to access YWD in addition to user name and password."		
**5-viii) Mode of delivery, features/functionalities/components of the intervention and comparator, and the theoretical framework**		
Please, refer to a description of YWD in the Introduction section.<br>Further description of YWD, see Multimedia Appendix.<br>The development of YWD is detailed in the reference.		
**5-ix) Describe use parameters**		
"Young people were encouraged to use YWD as a stand-alone resource and in collaboration with their parents and health care providers. They received no prompts to use YWD."		
**5-x) Clarify the level of human involvement**		
"Physicians, nurses, and dieticians provided the YWD intervention as part of usual outpatient care and saw participants from both the YWD and control groups. No extra time was allocated for the YWD intervention."<br><br>A number of health care providers from each setting is given in Multimedia Appendix 2.		
**5-xi) Report any prompts/reminders used**		
"They received no prompts to use YWD."		
**5-xii) Describe any co-interventions (incl. training/support)**		

"Participants were digitally randomized ... either to YWD and usual care (YWD group) or usual care alone (control)."<br><br>"Young people and parents downloaded YWD on their smartphone or tablet during a 10-minute initial face-to-face or telephone guidance session"		
**6a) CONSORT: Completely defined pre-specified primary and secondary outcome measures, including how and when they were assessed**		
The section "Outcome Measures" describes the primary and secondary outcomes including when and how they were assessed.		
**6a-i) Online questionnaires: describe if they were validated for online use and apply CHERRIES items to describe how the questionnaires were designed/deployed**		
"The psychometric scales, sociodemographic items, and YWD-specific questions were compiled into an electronic questionnaire. Face validity was tested in six young people before the trial start; no changes were required."		
**6a-ii) Describe whether and how "use" (including intensity of use/dosage) was defined/measured/monitored**		
"YWD users were defined as those who had used YWD on at least five days. The cutoff of five days was set to be sure the participants used the app more than the four times where they were paid a visit from the data collector (baseline, 2, 7, and 12 months). YWD use was documented by log data as time, date, and action (view, update, create, delete). Page hits were defined as the number of "clicks" within a function."		
**6a-iii) Describe whether, how, and when qualitative feedback from participants was obtained**		
A reference to a qualitative study (individual interviews) exploring the influence of YWD on self-management.		
**6b) CONSORT: Any changes to trial outcomes after the trial commenced, with reasons**		
"Data collected at "young peoples' choice of place and time of day"."		
**7a) CONSORT: How sample size was determined**		
**7a-i) Describe whether and how expected attrition was taken into account when calculating the sample size**		
"To compensate for potential dropouts, a 25% adjustment was made, resulting in a target sample size of 65 subjects per group."		
**7b) CONSORT: When applicable, explanation of any interim analyses and stopping guidelines**		
The section "Outcome Measures" describes the primary and secondary outcomes including when and how they were assessed.		
**8a) CONSORT: Method used to generate the random allocation sequence**		
"Participants were digitally randomized in a 1:1 allocation ratio either to YWD and usual care (YWD group) or usual care alone (control). They were stratified by department in random permuted blocks of two and four. "		
**8b) CONSORT: Type of randomisation; details of any restriction (such as blocking and block size)**		
"...stratified by department in random permuted blocks of two and four."		
**9) CONSORT: Mechanism used to implement the random allocation sequence (such as sequentially numbered containers), describing any steps taken to conceal the sequence until interventions were assigned**		
"Participants were digitally randomized..."		
**10) CONSORT: Who generated the random allocation sequence, who enrolled participants, and who assigned participants to interventions**		
"...digitally randomized."<br><br>"If young people were interested, a one-hour meeting was scheduled to complete written consents and randomization... After randomization, young people and parents downloaded YWD on their smartphone or tablet during a 10-minute initial face-to-face or telephone guidance session provided by the first author."		
**11a) CONSORT: Blinding - If done, who was blinded after assignment to interventions (for example, participants, care providers, those assessing outcomes) and how**		
**11a-i) Specify who was blinded, and who wasn't**		
"Blinding was not possible."<br><br>"Analyses were performed by a statistician blinded to group assignment ..."		
**11a-ii) Discuss e.g., whether participants knew which intervention was the "intervention of interest" and which one was the "comparator"**		
"Blinding was not possible."		

**11b) CONSORT: If relevant, description of the similarity of interventions**		
Not relevant		
**12a) CONSORT: Statistical methods used to compare groups for primary and secondary outcomes**		
The statistical methods used to compare groups for primary and secondary outcomes are described in the Statistical Analysis section.<br><br>"Due to stratified randomization, the department was included in the regression model as a categorical covariate."		
**12a-i) Imputation techniques to deal with attrition / missing values**		
All participants were included in the analysis.		
**12b) CONSORT: Methods for additional analyses, such as subgroup analyses and adjusted analyses**		
"The effect of YWD depends on use. Consequently, the CONSORT-EHEALTH checklist [21] recommends a sub-group analysis comparing users with nonusers, equivalent to an as-treated analysis. YWD use is a post-randomization variable, and the possibility that several unmeasured factors affected both the probability of noncompliance with the intervention and glycemic control confounds the as-treated analysis. We, therefore, focused on estimating the complier average causal effect of YWD [22]."		
RESULTS		
**13a) CONSORT: For each group, the numbers of participants who were randomly assigned, received intended treatment, and were analysed for the primary outcome**		
Described in details in the first section of Results and in Figure 1 (participant flow chart)		
**13b) CONSORT: For each group, losses and exclusions after randomisation, together with reasons**		
Figure 1 (participant flow chart)		
**13b-i) Attrition diagram**		
Figure 3. Number of young people who used YWD during the study.		
**14a) CONSORT: Dates defining the periods of recruitment and follow-up**		
"Young people and parents were recruited from November 2015 to March 2016."<br><br>"Outcomes data were collected at baseline and two months, seven months, and 12 months after YWD use began."		
**14a-i) Indicate if critical "secular events" fell into the study period**		
"Four major platform-specific technical issues occurred and were resolved: (1) January 2016, Android. Starting carbohydrate-counting-quiz resulted in log-off (duration=10 days, n=1), (2) March 2016, iOS. YWD could not open on some iPhone-software versions. Required re-installation (duration=10 days, n=7), (3) September 2016, Android. Unable to upload photos (duration=40 days, n=1), and (4) January 2017, iOS. YWD could not open due to update. Needed re-installation (duration=10 days, n=14)."		
**14b) CONSORT: Why the trial ended or was stopped (early)**		
Not relevant		
**15) CONSORT: A table showing baseline demographic and clinical characteristics for each group**		
Table 1. Sample characteristics		
**15-i) Report demographics associated with digital divide issues**		
Please, find details in Table 1. Sample characteristics		
**16a) CONSORT: For each group, number of participants (denominator) included in each analysis and whether the analysis was by original assigned groups**		
**16-i) Report multiple "denominators" and provide definitions**		

"YWD users were defined as those who had used YWD on at least five days."<br><br>"A total of 53 (70%) young people and 19 (39%) parents used YWD<br>on at least 5 days ..."<br><br>Please, refer to the Result section for further details.		
**16-ii) Primary analysis should be intent-to-treat**		
Please, refer to the Result section for details about the intention-to-treat analysis and the as-treated analysis, as well as the complier average causal effect of YWD.		
**17a) CONSORT: For each primary and secondary outcome, results for each group, and the estimated effect size and its precision (such as 95% confidence interval)**		
Please, refer to the Result section and Table 2 (Between-group differences in outcomes).		
**17a-i) Presentation of process outcomes such as metrics of use and intensity of use**		
"YWD use was documented by log data as time, date, and action (view, update, create, delete). Page hits were defined as the number of “clicks” within a function."<br><br>Figure 3. Number of young people who used YWD during the study.<br><br>Multimedia appendices describing the use of the chat room, the main functions and popular information topics.<br><br>Please, refer to the Results section (Young with Diabetes use) for further description.		
**17b) CONSORT: For binary outcomes, presentation of both absolute and relative effect sizes is recommended**		
Please, refer to the results section.		
**18) CONSORT: Results of any other analyses performed, including subgroup analyses and adjusted analyses, distinguishing pre-specified from exploratory**		
Please, refer to the results section		
**18-i) Subgroup analysis of comparing only users**		
Subgroup analyses are performed. Please, refer to the results section.		
**19) CONSORT: All important harms or unintended effects in each group**		
Not relevant		
**19-i) Include privacy breaches, technical problems**		
"Four major platform-specific technical issues occurred and were resolved: (1) January 2016, Android. Starting carbohydrate-counting-quiz resulted in log-off (duration=10 days, n=1), (2) March 2016, iOS. YWD could not open on some<br>iPhone-software versions. Required re-installation (duration=10 days, n=7), (3) September 2016, Android. Unable to upload photos (duration=40 days, n=1), and (4) January 2017, iOS. YWD could not open due to update. Needed re-installation<br>(duration=10 days, n=14). In addition, participants reported minor technical issues, such as having lost the YWD app due to new or broken phones (young people=26, parents= 2). A total of 43 (57%) young people and eight (16%) parents reported technical issues."		
**19-ii) Include qualitative feedback from participants or observations from staff/researchers**		
The paper includes a reference to a qualitative study (individual interviews) exploring the influence of YWD.		
DISCUSSION		
**20) CONSORT: Trial limitations, addressing sources of potential bias, imprecision, multiplicity of analyses**		
**20-i) Typical limitations in ehealth trials**		
Please, refer to limitations		
**21) CONSORT: Generalisability (external validity, applicability) of the trial findings**		

**21-i) Generalizability to other populations**		
**21-ii) Discuss if there were elements in the RCT that would be different in a routine application setting**		
**22) CONSORT: Interpretation consistent with results, balancing benefits and harms, and considering other relevant evidence**		
**22-i) Restate study questions and summarize the answers suggested by the data, starting with primary outcomes and process outcomes (use)**		
Please, refer to the beginning of the Discussion section.		
**22-ii) Highlight unanswered new questions, suggest future research**		
Please, refer to the Discussion section		
Other information		
**23) CONSORT: Registration number and name of trial registry**		
Trial registration is described in the abstract and methods section		
**24) CONSORT: Where the full trial protocol can be accessed, if available**		
Not published. The study is registered at ClinicalTrials.gov.		
**25) CONSORT: Sources of funding and other support (such as supply of drugs), role of funders**		
"This study was funded by Danish Agency for Digitisation, Centre for Telemedicine, Capital Region of Denmark and a Research Grant from Nordsjællands Hospital, Hillerød."		
**X26-i) Comment on ethics committee approval**		
"The study was approved by the Danish Data Protection Agency (no. 04015<br>NOH-2015-031) and performed in accordance with ethical recommendations of Helsinki Declaration. Ethical approval by Research Ethics Committee was not necessary (Ref.no. 14013934)."		
**x26-ii) Outline informed consent procedures**		
"Written informed consent was obtained from young people and parents, and parental consent was required for participants younger than 18 years."<br><br>"... a one-hour meeting was scheduled to complete written consents and randomization."		
**X26-iii) Safety and security procedures**		
"YWD complies with regulations for protecting personal health information. A code was required to access YWD in addition to user name and password."<br><br>"...a telephone hotline was available for technical difficulties."<br><br>"Health care providers attended YWD training:..."<br><br>"... young people and parents downloaded YWD on their smartphone or tablet during a 10-minute initial face-to-face or telephone guidance session..."		
**X27-i) State the relation of the study team towards the system being evaluated**		
"YWD was developed in cooperation with the IT enterprise Mobile Fitness A/S and the project group (including the authors). The project group owns the national rights."		
